# Age effects on plasma cholesterol and triglyceride profiles and metabolite concentrations in dogs

**DOI:** 10.1186/1746-6148-10-57

**Published:** 2014-03-05

**Authors:** Koh Kawasumi, Nanae Kashiwado, Yuki Okada, Masaki Sawamura, Yasuhiro Sasaki, Eiji Iwazaki, Nobuko Mori, Ichiro Yamamoto, Toshiro Arai

**Affiliations:** 1 Department of Veterinary Science, School of Veterinary Medicine, Nippon Veterinary and Life Science University, 1-7-1 Kyonancho Musashino, Tokyo 180-8602, Japan; 2 Sawamura Veterinary Hospital, 6-37 Higashikamijyuku, Togane, Chiba 283-0067, Japan; 3 Celebre Animal Hospital, Pacific squre Bld. 1F, 3-1-11 Yoyogi, Shibuya-ku, Tokyo 151-0053, Japan

**Keywords:** Aged dog, Adiponectin, Superoxide dismutase, Alpha1-acid glycoprotein, Cholesterol lipoprotein, Triglyceride lipoprotein

## Abstract

**Background:**

In dogs, occurrence of lipid metabolism disorders such as obesity and diabetes mellitus has increased markedly in recent years. Hyperlipidemia has been regarded as a common characteristic for obese animals and hyperlipidemic condition may be associated with inflammation, oxidative stress and lipid composition changes. In this study, we investigated the changes in plasma cholesterol and triglyceride (TG) profiles and metabolite concentrations in 24 dogs (young group: 0-7 years old, n = 12, aged group: 8-13 years old, n = 12).

**Results:**

Plasma adiponectin (ADN) concentrations were significantly lower in aged dogs than those in young dogs (mean ± SD, 17.2 ± 10.0 μg mL^-1^ vs 29.3 ± 12.5 μg mL^-1^, respectively; P <0.05). Although there were no significant differences statistically, aged dogs showed significantly higher plasma alpha1- acid glycoprotein (alpah1-AG) levels compared to those in young dogs. Plasma cholesterol lipoprotein and TG lipoprotein were divided into four fractions by biphasic agarose gel electrophoresis technique. The levels of the third TG-lipoprotein fraction from the positive pole (TG Fraction 3) were significantly higher in aged dogs than in young dogs (mean ± SD, 143.0 ± 109.3 mg dL^-1^ vs 55.2 ± 31.3 mg dL^-1^, respectively; P <0.05). On the correlation coefficient analysis by Peason’s method, moderate positive correlations were seen between the age and TG (r = 0.446, P = 0.029), TG Fraction 3 (r = 0.516, P = 0.010), malondialdehyde (r = 0.146, P = 0.043), alpha-1 AG (r = 0.448, P = 0.028) levels, respectively. Moderate negative correlations were seen the age and total cholesterol (TC) Fraction 2 (r = -0.446, P = 0.029), glucose (r = -0.637, P = 0.001), ADN (r = -0.408, P = 0.048), respectively.

**Conclusions:**

Present data suggest biochemical characteristics of lipid metabolism disorder may be affected by aging in dogs.

## Background

Hyperlipidemia has been regarded as a common characteristic for obese dogs [1], and prevalence of overweight and obese dog has been estimated as 24% to 30% [2]. Hyperlipidemic condition could be a deteriorative factor for metabolic syndrome in dogs [3]. Physiological variables, such as age, body weight and sex may affect hyperlipidemia severity in dogs [4]. Effect of aging on plasma total cholesterol and triglyceride levels in hyperlipidemic dogs has been reported by our colleagues [5]. We have to pay much attention to lipid metabolic condition associated with lipid oxidative stress, antioxidant and inflammation degree, and insulin resistance in dogs with different ages. Hyperlipidemia in aged dogs seems to be confounded by nutrition states, inflammation, and oxidative stress as the same as in aged human [6].

Plasma malondialdehyde (MDA) concentrations have been regarded as a lipid peroxide stress marker [7], and the correlation between plasma lipid peroxide levels and high prevalence of diabetic patients has been reported in human [8,9]. Superoxide dismutase and glutathione peroxidase have been regarded as enzymes that increase with oxidation stress elevation. Alpha 1-acid glycoprotein is an acute phase protein that is produced in the liver in response to inflammation. Excess non-esterified fatty acid (NEFA) released from adipocytes induces lipotoxicity. Plasma insulin and adiponectin are hormones that suggest the degree of insulin resistance. In this study, we investigated the changes in above mentioned plasma parameters and cholesterol and TG profiles in 24 dogs with different ages though we could not elucidate the daily rhythmicity of lipid levels completely [10].

## Methods

### Animals

Twelve client-owned young dogs (female *n* = 7, male *n* = 5, 0-7 years old) and twelve aged dogs (female *n* = 7, male *n* = 5, 8-13 years old) were examined. The age, breed, sex and body weight of dogs used in the present study were shown in Table 1. Each dog was given a meal twice a day. The diet composition varied among examined dogs, since each dog’s owner was permitted to give normal diet in this study. All animals exhibited no clinical signs for diseases and were not on any medication at the time of the study. They were recommended to examine plasma biomarker levels on lipid metabolism by veterinarians. Any clinically treated animals were excluded from the study. The degree of obesity in dogs was assessed by a five-level body conditioning score (BCS) as follows [1-5]: very thin [1], underweight [2], ideal [3], over weight [4] and obese [5], commonly used in Japan [1]. Ethical approval was obtained from the Nippon Veterinary and Life Science University Animal Research Committee (No.13-92). Written informed consent was obtained from the owners of the animals in this study.

**Table 1:** Profile of the age, breed, sex and body weight (BW) in young and aged dogs

	**No.**	**Age (years old)**	**Breed**	**Sex**	**BW (kg)**
A	1	0	Toy poodle	F	5.5
	2	0	Mix	M	19
	3	1	Miniature pinscher	M	4.5
	4	2	Miniature pinscher	M	4.3
	5	3	Miniature dachshund	F	2.8
	6	3	German shepherd	F	25.0
	7	4	Belgian tervuren	M	23.2
	8	5	Miniature dachshund	F	5.8
	9	5	Miniature dachshund	F	5.6
	10	5	Pekingese	F	4.4
	11	7	Toy poodle	M	3
	12	7	Miniature schnauzer	F	5.6
B	1	8	Miniature schnauzer	M	6.0
	2	8	Miniature dachshund	M	4.3
	3	8	Beagle	M	16.7
	4	8	Miniature dachshund	F	5.5
	5	8	Toy poodle	F	3.9
	6	9	Shih tzu	M	10.0
	7	9	Miniature schnauzer	M	7.1
	8	10	Miniature dachshund	F	5.8
	9	10	Miniature schnauzer	F	5.6
	10	12	Toy poodle	F	2.72
	11	13	Pomeranian	F	5.3
	12	13	Miniature dachshund	F	4.8

A: young group (0-7 years old).

B: aged group (8-13 years old).

Blood samples were taken from jugular veins of dogs fasted overnight (without any nutrient for over 8 hours after the last meal) in heparinized tubes. Plasma was recovered by centrifugation at 3,000 rpm, for 15 min at 4°C in each veterinary clinic and subsequently stored at -25°C until use. Glucose (GLU), TC, TG, total protein (TP), blood urea nitrogen (BUN) and creatinine (CRE) concentrations and alanine aminotransferase (ALT), aspartate aminotransferase (AST), alkaline phosphatase (ALP) activities were measured using an autoanalyzer (JCA-BM2250, JEOL Ltd., Tokyo, Japan) with the manufacture’s reagents at Monolis Inc. (Tokyo, Japan). It took less than a week to obtain the results of laboratory analysis.

Plasma alpha1-acid glycoprotein (alpha1-AG) concentrations were measured by single radial immunodiffusion method at Monolis Inc. Plasma NEFA and MDA concentrations were measured using commercial kit, NEFA-C test (Wako Pure Chemical Industries, Inc., Tokyo, Japan), and NWLSS^TM^ Malondialdehyde assay (Northwest Life Science Specialties, LLC, Vancouver, Canada), respectively. Plasma insulin (INS) and adiponectin (ADN) concentrations were measured with commercial ELISA kits, Lbis dog insulin kit (SHIBAYAGI Co., Gunma, Japan), mouse/rat adiponectin kit (Otsuka Pharmaceutical Co., Ltd, Tokyo, Japan), respectively. Plasma SOD activity and GSHPx activities were measured using commercial kit, NWLSS^TM^ Superoxide Dismutase Activity Assay (Northwest Life Science Specialties, LLC, Vancouver, Canada), and NWLSS^TM^ Glutathione Peroxidase Assay (Northwest Life Science Specialties, LLC, Vancouver, Canada), respectively.

Plasma lipoprotein cholesterol and TG profiles were measured by the biphasic agarose gel electrophoresis method using commercial kit, Quickgel LIPO gels (Helena Laboratories, Saitama, Japan) with Cho/Trig COMBO reagents (Helena Laboratories, Saitama, Japan).

### Statistical analysis

Results are presented as mean ± SD. Statistical significance was determined by unpaired Student’s t-test. The significance level was set at P < 0.05.

Correlation coefficient between the age and each parameter value was calculated by Peason’s method. The significance level was set at P < 0.05*, P* < 0.01, respectively.

## Results

**Figure 1:**
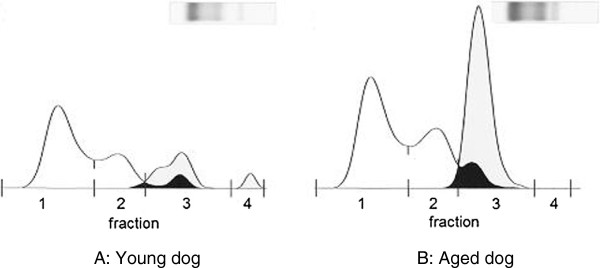
**Gel electrophoresis and profiles for cholesterol lipoprotein and triglyceride lipoprotein. (A)** young dog and **(B)** aged dog. White zones; cholesterol lipoprotein fractions Gray zones; triglyceride lipoprotein fractions. Black zones; cholesterol lipoprotein fractions and triglyceride lipoprotein fractions are piled up.

As shown in Table 2, BCS (mean ± SD) in young dogs was 2.8 ± 0.7, while that in aged dogs was 3.4 ± 0.8. Though there were no significant differences statistically, plasma TG, MDA and NEFA levels in aged dogs were higher than those in young dogs. Using electrophoretic measurements, the level of the third TG-lipoprotein fraction from the positive pole (Fraction 3) in aged dogs was determined significantly higher than that in young animals. (mean ± SD, 143.0 ± 109.3 mg dL^-1^ vs 55.2 ± 31.3 mg dL^-1^, respectively; *P* <0.05). Representative cholesterol lipoprotein and TG lipoprotein electrophoresis tracing in young and aged dogs were shown in Figure 1A and B, respectively. The biphasic agarose gel electrophoresis technique revealed a prominent third TG-lipoprotein fraction from the positive pole (Fraction 3) in aged dogs.

**Table 2:** Plasma TC, TG, lipoprotein compositions, MDA, and NEFA concentrations in dogs with aging

	**0-7 years (12)**	**8-13 years (12)**
BCS	2.8 ± 0.7	3.4 ± 0.8
TC (mg dL-1)	284.8 ± 111.2	229.3 ± 87.4
Fraction 1 (mg dL-1)	192.2 ± 47.5	175.4 ± 45.7
Fraction 2 (mg dL-1)	79.8 ± 72.6	27.4 ± 15.6 (11)*
Fraction 3 (mg dL-1)	11.7 ± 6.6	15.8 ± 14.8
Fraction 4 (mg dL-1)	3.6 ± 3.3 (4)	0 (11)*
TG (mg dL-1)	87.8 ± 68.0	160.2 ± 119.7
Fraction 1 (mg dL-1)	0	0
Fraction 2 (mg dL-1)	2.2 ± 1.7 (8)	0.5 ± 0.5 (7)*
Fraction 3 (mg dL-1)	55.2 ± 31.3	143.0 ± 109.3*
Fraction 4 (mg dL-1)	41.8 ± 36.4 (6)	47.8 ± 16.2 (4)
MDA (μm ol L-1)	4.0 ± 1.5	4.9 ± 2.2
NEFA (mEq L-1)	0.7 ± 0.3	0.9 ± 0.3

The numbers in parentheses indicate the number of animals examined.

Data are expressed as mean ± SD.

*Significant (P < 0.05) when compared against 0-7 years old group.

As shown in Table 3, plasma AST, ALP activities, INS concentrations and plasma alpha1-AG levels in aged dogs were higher than those in young animals, though there were no significant differences. Plasma SOD activitity in aged dogs was significantly higher than that in young dogs (mean ± SD, 28.0 ± 13.3 U mL^-1^ vs 10.8 ± 2.6 U mL^-1^, respectively; P <0.05). Plasma ADN concentration in aged dogs was significantly lower than that in young dogs (mean ± SD, 17.2 ± 10.0 μg mL^-1^ vs 29.3 ± 12.5 μg mL^-1^, respectively; P < 0.05). There were no significance differences seen in plasma GSHPx activities.

**Table 3:** Plasma GLU, TP, AST, ALT, ALP, BUN, CRE, INS, ADN, SOD, alpha1-AG concentrations in dogs with aging

	**0-7 years (12)**	**8-13 years (12)**
GLU (mg dL-1)	106.7 ± 15.7	89.0 ± 12.0*
TP (g dL-1)	6.9 ± 0.5	7.3 ± 0.7
AST (IU L-1)	32.3 ± 8.5	39.1 ± 26.0
ALT (IU L-1)	51.4 ± 29.4	50.2 ± 19.6
ALP (IU L-1)	124.3 ± 64.5 (11)	230.6 ± 117.6 (10)
BUN (mg dL-1)	22.6 ± 9.8	17.7 ± 6.7
CRE (mg dL-1)	1.1 ± 0.2	1.0 ± 0.2
INS (ng mL-1)	0.9 ± 0.3	1.5 ± 1.0
ADN (μg mL-1)	29.3 ± 12.5	17.2 ± 10.0*
SOD (U mL-1)	10.8 ± 2.6 (6)	28.0 ± 13.3 (7)*
GSHPx (mU mL-1)	53.3 ± 11.6	54.8 ± 10.9 (9)
alpha1-A G (μg mL-1)	90.9 ± 77.1	172.9 ± 109.8

The numbers in parentheses indicate the number of animals examined.

Data are expressed as mean ± SD.

*Significant (P < 0.05) when compared against 0-7 years old group.

As shown in Table 4, the correlation coefficients were calculated by Peason’s method. Moderatepositive correlations were seen between the age and TG (r = 0.446, P = 0.029), TG Fraction 3 (r = 0.516, P = 0.010), MDA (r = 0.146, P = 0.043), alpha-1 AG (r = 0.448, P = 0.028) levels, respectively. Moderate negative correlations were seen between the age and cholesterol lipoprotein Fraction 2 (r = -0.446, P = 0.029), glucose (r = -0.637, P = 0.001), ADN (r = -0.408, P = 0.048), respectively.

**Table 4:** Correlation coefficients and P value between the age and examined parameters in 24 dogs

**Correlation**	**Coefficient**	**P value**
BCS	0.260	0.219
BW	- 0.314	0.135
TC	- 0.362	0.083
Fraction 1	- 0.255	0.23
Fraction 2	- 0.446	0.029*
Fraction 3	0.191	0.372
Fraction 4	- 0.054	0.803
TG	0.446	0.029
Fraction 1	- 0.014	0.95
Fraction 2	- 0.110	0.607
Fraction 3	0.516	0.010 **
Fraction 4	- 0.028	0.98
MDA	0.416	0.043*
NEFA	0 .135	0.53
GLU	- 0.637	0.001 **
TP	0 .2 1 1	0.322
AST	0.162	0.449
ALT	- 0.129	0.548
ALP	0.304	0.148
BUN	- 0.336	0.109
RE	- 0.287	0.174
INS	0 159	0.459
ADN	- 0.408	0.048*
SOD	0 203	0.342
G SHPx	- 0.034	0.876
alph1a- AG	0.448	0.028*

Correlation coefficients w ere caliculated by Peason's method.

*Significan celevel wass etat P < 0.05.

**Signific an c eleve l was set a t P < 0.01.

## Discussion

In this study, 4 Miniature Schnauzer were included. However, they will need to be separated from other breeds for further study since they have genetic problems associated with lipid metabolism. BCS in aged dogs was higher than that in young dogs and plasma lipid metabolic biomarker, TG, MDA, and NEFA levels increased with aging. In addition, moderate positive correlations (correlation coefficient: 0.4-0.7) were seen between the age and TG, TG Fraction 3, MDA, alpha1-AG levels, respectively, while moderate negative correlations (correlation coefficient:-0.4- -0.7) were seen between the age and cholesterol lipoprotein Fraction 2, GLU, ADN levels, respectively by Peason’s analysis.

It was reported that increase in plasma TG levels was one of characteristics indicating lipid accumulation in the liver of animals [11]. On the contrary, plasma ADN concentrations significantly decreased in aged dog group with a higher mean BCS compared to the young dog group. It was reported that adipose cells secreted decreased amounts of ADN as lipid accumulated in subcutaneous and visceral portion of the body [12].

Generally, plasma levels of MDA as a lipid peroxide stress marker increase in presence of oxidative stress. It was reported that serum MDA levels in cancer dogs with oxidative stress were significantly higher than those in clinically normal dogs [13]. Increase in plasma MDA concentrations in aged dogs may indicate increase in lipid peroxide stress in aged dogs with tendency to be overweight. In addition, plasma MDA levels are considered to be lipid peroxide stress marker that may suggest early stage of lipid metabolic disorder in dogs. Plasma NEFA levels in aged dogs were higher than those in young dogs. It was reported that increased plasma NEFA induced heterotopy lipid accumulation in the body and excess β-oxidation in pancreatic β-cells, which induced reactive oxygen species (ROS) generation [14]. Finally, excess NEFA induced pancreatic β cell dysfunction, which referred to as lipotoxicity [15,16]. Lipotoxicity induces insulin resistance [17]. Plasma INS concentrations in aged dogs were higher than those in young dogs, which may suggest an association between lipid metabolic disorder and insulin resistance with aging in dogs.

The activities of plasma SOD, an antioxidant, were higher in aged dogs. It is speculated that one of reasons that plasma antioxidant increase with aging was due to the lipid peroxide increase with aging. In the present study, plasma MDA levels were higher in aged dogs. Plasma SOD levels in 6 young dogs and 7 aged dogs were analyzed statistically, since 6 of 12 in young dogs and 5 of 12 in aged dogs had SOD levels lower than detectable range under detection limit levels (<5 U mL^-1^), while plasma GSHPx levels in 9 aged dogs were analyzed statistically, since 3 of 12 in aged dogs indicated above detection limit levels (> 65 mU L^-1^).Plasma alpha1-AG levels in aged dogs were significantly higher than those in young dogs. These results suggest that inflammatory condition associated with lipid accumulation may progress in aged dogs. Acute phase proteins will play an important role at early stage of lipid disorder in dogs. The third TG-lipoprotein fraction from the positive pole (Fraction 3) in aged dogs was prominent as revealed by the biphasic agarose gel electrophoresis technique (Figure 1B). We think this fraction may be very low-density lipoprotein-triglyceride. Further comparative examination on TG lipoprotein fractions separated by electrophoresis versus ultracentrifugation will be necessary to confirm this point.

In our previous report [18], we proposed the new criteria to detect canine hyperlipidemia at early stage based on the any two of the following three factors, namely elevated plasma triglyceride (TG) (≥165 mg dL^-1^), total cholesterol (TC) (≥200 mg dL^-1^) and non-esterified fatty acid (NEFA) (≥1.5 mEq L^-1^) levels. Based on the criteria, 1 of 12 (8.3%) in young dogs and 4 of 12 (33.3%) in aged dogs were diagnosed as hyperlipidemia in the present study.

## Conclusions

Lipid metabolic disorder seems to be complicated by nutrition states, inflammation, oxidative stress in dogs. Present data suggested that these factors were affected by aging. We should continue further examination to clarify these relationships in order to confirm its reliability in detecting early stage of lipid metabolic disorder in dogs.

## Abbreviations

alpha1-AG: alpha1-acid glycoprotein; ADN: Adiponectin; ALP: Alkaline phosphatase; ALT: Alanine aminotransferase; AST: Aspartate aminotransferase; BCS: Body condition score; BUN: Blood urea nitrogen; CRE: Creatinine; GLU: Glucose; GSHPx: Glutathione peroxidase; MDA: Malondialdehyde; NEFA: Non-esterified fatty acid; ROS: Reactive oxygen species; SOD: Superoxide dismutase; TC: Total cholesterol; TG: Triglyceride; TP: Total protein.

## Competing interests

The authors declare that they have no competing interests.

## Authors’ contributions

TA designed the study and approved the manuscript. KK, NK, YO, EI, NM and IY analyzed data and KK prepared the manuscript. MS and YS collected experimental samples. All authors read and approved the final manuscript.
